# Editorial: Mental health in the age of artificial intelligence

**DOI:** 10.3389/fpsyt.2025.1750256

**Published:** 2026-01-06

**Authors:** Jasmine M. Noble, Kyooseob Ha, Andrew James Greenshaw

**Affiliations:** 1 Computational Psychiatry Group, Department of Psychiatry, Faculty of Medicine and Dentistry, University of Alberta, Edmonton, AB, Canada; 2 Department of Psychiatry, Division of Neuroscience and Translational Psychiatry, University of British Columbia, Vancouver, BC, Canada; 3 Department of Psychiatry, Faculty of Medicine and Dentistry, University of Alberta, Edmonton, AB, Canada

**Keywords:** artificial intelligence, conversational agent, chatbot, mental health, psychiatry, digital mental health, large language model, ethical AI

## Introduction

Artificial intelligence (AI) is rapidly maturing, with applications spanning the full spectrum of healthcare, from foundational research to clinical implementation. Its use has extended beyond lab-based proofs of concept into real-world settings (1), including but not limited to clinical documentation (2, 3), administrative triage (4, 5), diagnostic support (6), and conversational agents (7, 8). These innovations hold the potential to expand access to care (9, 10), personalize treatment (7, 11–13), predict risk (14, 15), and ease the administrative burdens (2, 4) on strained systems.

Despite the global prevalence and economic burden of mental health conditions (16), the clinical adoption of AI in the field of psychiatry remains limited (13). This lag reflects the intrinsic complexity of the field, which challenges conventional algorithmic approaches and underscores the need for AI solutions that are ethically grounded, reproducible, contextually adaptive, and attuned and supportive to the nuances of human experience.

The Research Topic, “*Mental Health in the Age of Artificial Intelligence*”, explores this rapidly shifting terrain through five timely contributions. Each publication offers a unique lens, spanning methodological, empirical, cultural, and existential dimensions, with ethical reflection as a common, unifying thread. Together they converge on a single, urgent imperative: for AI to be more widely trusted and in turn adopted in the mental health ecosystem, it must be designed, deployed, and evaluated in ways that are accountable, reproducible, fair, and deeply human-centered.

These themes surface immediately in the first contribution. The question of reproducibility and interpretability has become a cornerstone of responsible AI (13, 17), and Celeste et al. tackle it head-on in their contribution, “*A software pipeline for systematizing machine learning of* sp*eech data*”. Here they offer a suite of configurable software pipelines built within Python Luigi which are then used to test the reproducibility of three machine learning studies, involving depression, mild cognitive impairment, and aphasia, as a proof of concept. Authors then warn of the reproducibility crisis and argue that the ability to reproduce machine learning experiments, including model configurations, optimal hyperparameters, validation predictions, and performance metrics, is not merely a methodological ideal, but a moral and professional responsibility.

The ethical conversation then shifts to raise the spotlight on the human experience. Lee et al in “Artificial intelligence conversational agents in mental health: Patients see potential, but prefer humans in the loop,” gathers feedback from patients with self-reported mild to moderate anxiety in terms of their experiences, perceptions, and acceptability of mental health AI conversational agents (CAs). This timely study poses a clinically meaningful question at a critical juncture in the adoption of digital mental health tools, highlighting the importance of amplifying the patient’s voice within a values-based framework that is increasingly recognized as best practice (13, 18). The authors findings remind us while there are perceptions of utility and benefit in terms of potential increases in access to care through use of AI chatbots, the perceived risk of lack of empathy, concerns over privacy and other technical limitations of these models remain leading concerns, and participants signalled a consistent preference for “human-in-the-loop” models wherein AI serves as an extender of care, not a replacement for it. This notion is reinforced in a recent review, where while AI-based CAs were found to be more effective for clinical and subclinical populations, the need persists to “untangle the complex interplay” between a variety of factors, including when “human support is indispensable.” (19).

The ethical conversation continues in, Denecke and Gabarron’s “The ethical aspects of integrating sentiment and emotion analysis in chatbots for depression intervention,” where authors explore the ethical dimensions of sentiment analysis in chatbots seeking to support individuals with the provision of depression specific interventions. Reflecting the cautions of other authors in other works included in the special topic, the importance of balance is reiterated, as misclassification of emotion/harms increase risk of inappropriate or missed system responses, risk detection, and risk escalation. Authors emphasize the importance of thoughtfully integrating chatbots into care settings under the supervision of qualified health professionals. They stress that emotion should be treated as a complex, clinically significant, and ethically sensitive signal that demands careful and responsible handling. This reflects findings from other recent meta-analysis of AI-based CAs, where while a large effect size for AI-based CA is observed for the mitigation of psychological distress (especially when multi-modal), they can still generate “unnatural or repetitive interactions, potentially reducing clinical effectiveness.” (19).

Questions about acceptability and safety are magnified when AI systems are designed to generate text, make inferences, or interact dynamically with users. In “Applications of large language models in psychiatry: a systematic review,” Omar et al. synthesize a growing body of evidence on the use of large language models (LLMs) in mental health contexts. While they identify promising applications in clinical reasoning, educational tools, and even therapeutic support, the review also highlights critical issues in the underestimation of suicide risk, inconsistency in complex scenarios, and the lack of rigorous safety evaluation. Their findings align with emerging international concerns and caution in the field, such as declining medical safety messaging in generative AI models which are estimated to have reduced from ~26% to 1% over the last 2–3 years (20). While LLMs offer flexibility and scalability, their implementation should proceed cautiously and with significant oversight.

Finally, Alkhalifah et al. remind us to reflect on the human experience. In “Existential anxiety about artificial intelligence (AI): is it the end of humanity era or a new chapter in the human revolution?,” The authors explore public perceptions of AI’s role in society, and in particular, its psychological and existential consequences. Drawing on survey data from a public population in Saudi Arabia, they find significant levels of AI-related anxiety, including fears of human obsolescence, concerns of unpredictability, and sense of emptiness. The authors acknowledge this underlying unease as a sentiment that warrants attention and consideration, positioning it as pertinent to broader discussions about AI adoption, particularly its influence on social systems and its deeper implications for our understanding of what it means to be human.

Taken together, these five articles provide a cross-section of where the field stands, while offering an ethical and reflective pathway forward. Each surface that while the technological promise of AI is real and growing, its ethical, clinical, and human foundations require careful consideration. We must ensure systems are reproducible in their development, calibrated and fair in their outputs, interpretable in their logic and iterations, accountable in their consequences, and deeply and deliberately human-centered.

We must do all this while juggling the need to fully realize the potential of these innovations, while ensuring the appropriate safeguards are in place to protect end users. In a world arguably gripped by what Günther Anders’ described as “Promethean Shame,” a sense of human inadequacy in the face of our own technological creations, we find ourselves drawn to this promise to transcend our biological limits, even as we fail to grasp the full intended and unintended repercussions of these innovations, and the ways it unsettles deeply held values, purposes, and understandings of meaning (21).

At the same time, the accelerating momentum of commercial AI development, often obscured by proprietary opacity, is outpacing our existing systems for evaluation, governance, and ethical oversight. As this monopolized and monetized structure threatens to consolidate power further, we must confront a pressing question: on whose terms is mental health care evolving? Again, we see that the future of AI in this space is not solely a technical matter. It is also a clinical, philosophical, and political one, requiring sustained dialogue, shared standards, and a commitment to human-centered care.
